# Management of Bone Loss After Multiple Metacarpal and Distal Row Carpal Resection Due to Osteomyelitis

**DOI:** 10.7759/cureus.70186

**Published:** 2024-09-25

**Authors:** Leeann Qubain, Shaheryar Asad, Steve Miller, Karston Carr, Joshua W Hustedt

**Affiliations:** 1 Orthopedic Surgery, University of Arizona College of Medicine - Phoenix, Phoenix, USA; 2 Orthopedic Surgery, California Northstate University College of Medicine, Elk Grove, USA; 3 Orthopedic Surgery, OrthoArizona, Phoenix, USA

**Keywords:** delayed diagnosis, hand reconstruction, osteomyelitis treatment, wrist reconstruction, wrist septic arthritis

## Abstract

Atraumatic wrist pain can be due to a variety of causes including gout, pseudogout, cellulitis, arthritis flare, or infection of the joint. One important differential to rule out immediately is septic arthritis as it is considered an orthopedic emergency. Due to the rarity of septic arthritis in the wrist, there is limited data to guide diagnosis and treatment. Furthermore, delayed diagnosis of septic arthritis can progress to osteomyelitis and result in severe damage. The primary objective of this study is to present a case of atraumatic septic arthritis with a delayed diagnosis that developed into osteomyelitis in the left wrist and hand of an immunocompetent hand surgeon. In addition, we discuss the surgical treatment including reconstruction of the hand and wrist through a multidisciplinary approach.

## Introduction

Common causes of atraumatic wrist pain, erythema, and edema include gout, pseudogout, cellulitis, arthritis flare, or infection of the joint [1]. One important differential to rule out immediately is septic arthritis as it is considered an orthopedic emergency requiring urgent irrigation and debridement combined with parenteral antibiotics [2-4]. Distinguishing the cause of wrist pain among the multiple causes is challenging and critical. A misdiagnosis of septic arthritis can lead to devastating outcomes. For any infected joint, negative side effects such as cartilage destruction can occur within eight hours after infection and lead to permanent damage of the joint [5,6].

Clinical presentation among the various differential diagnoses is often inconclusive. To further complicate matters, septic arthritis can occur with simultaneous inflammatory arthropathy. Due to the rarity of septic arthritis in the wrist, there is limited data to guide diagnosis and treatment. There are no specific serum lab values that determine septic arthritis of the wrist [1,4,7,8]. Erythrocyte sedimentation rate (ESR), C-reactive protein (CRP), and wrist arthrocentesis with fluid analysis through cell count, gram stain, and culture can be used as tools to help in the diagnosis of septic arthritis.

However, Skeete et al. reviewed 804 patients with wrist pain and swelling. A total of 104 patients were suspicious for septic arthritis. Of the five patients with proven septic arthritis, four were immunocompromised and none of the patients had an above-average white blood cell count, CRP, or ESR compared to the other groups [1]. This emphasizes the importance of interpreting laboratory values as part of the decision process in diagnosing an infected wrist joint. Furthermore, in the same study, wrist arthrocentesis was attempted in all patients, and they were all subsequently diagnosed with infections. One aspiration out of five was unsuccessful and two of the five had insufficient fluid for analysis. Three of the five aspirations were not diagnostic for septic arthritis. This highlights the difficulty of diagnosing an infected wrist joint even with resources available [1].

Moreover, delaying the diagnosis of septic arthritis can lead to further damage of the bone and cartilage in the affected joint [9]. This further damage can lead to infection of the bone surrounding the affected joint and may result in the development of osteomyelitis. Osteomyelitis is an infection of the bone that typically occurs through hematogenous spread, contiguous spread from the surrounding tissue and joint, or direct inoculation of the bone from trauma or surgery [9]. The most common organism that results in chronic and acute osteomyelitis in adults is *Staphylococcus aureus* [9].

The primary objective of this study is to present a case of atraumatic septic arthritis with a delayed diagnosis that developed into osteomyelitis in the left wrist and hand of an immunocompetent hand surgeon. In addition, we discuss the surgical treatment including reconstruction of the hand and wrist through a multidisciplinary approach. 

## Case presentation

Patient history

**Figure 1:**
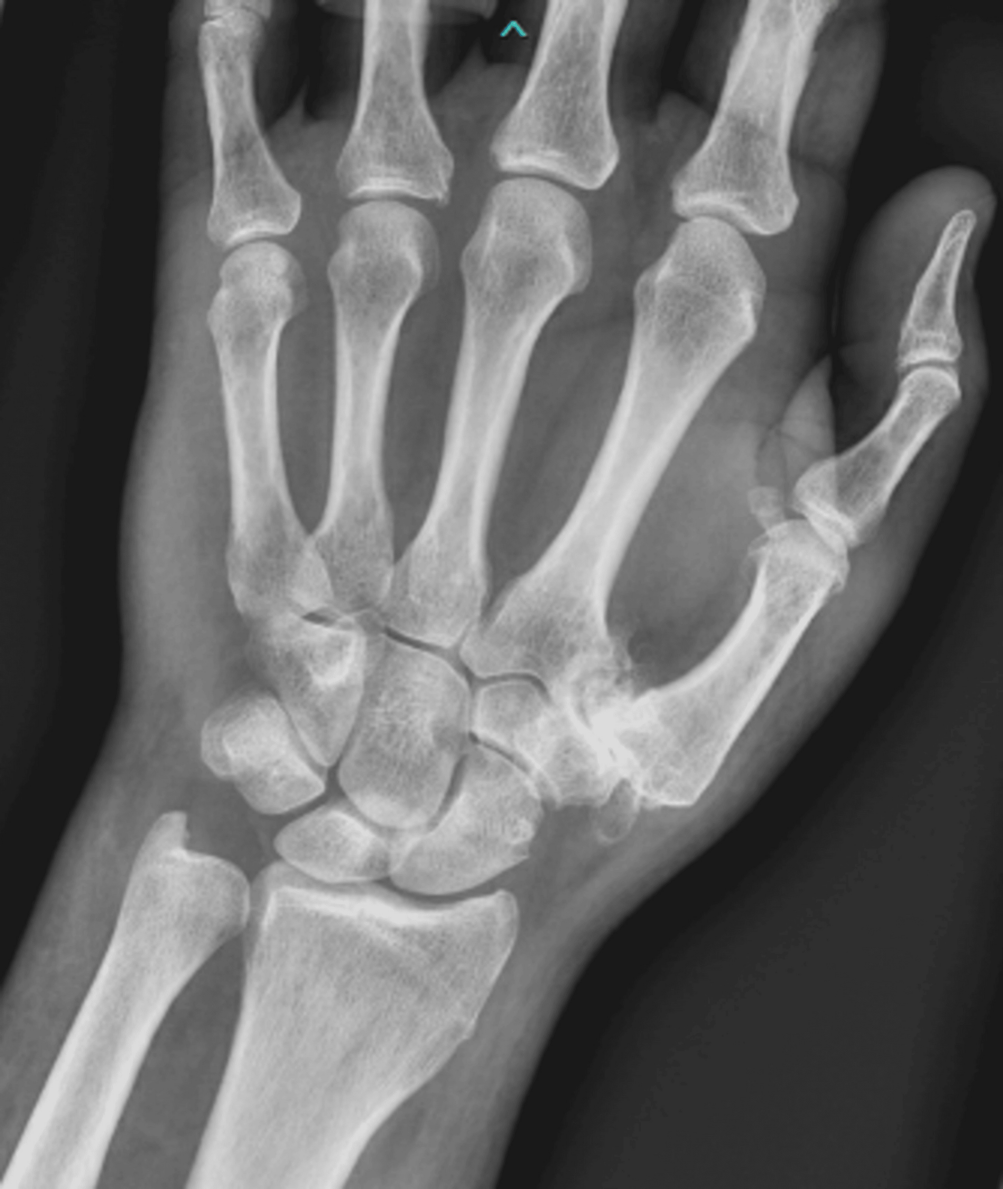
Initial left-hand radiograph.

A 68-year-old, right-hand dominant male with a past medical history of coronary artery disease, mild osteoarthritis, and nummular eczema presented with sudden onset left wrist pain and mild left wrist swelling with mild erythema. No fevers, chills, or history of recent trauma were reported. He also had no past medical history of diabetes mellitus or prior chemotherapy. Notably, he had received a COVID-19 booster two weeks prior to the presentation. He was seen by a rheumatologist within 48 hours, had a hand radiograph done (Figure 1) that showed carpometacarpal (CMC) arthritis, and was thought to have gout and was treated with a course of steroids and triamcinolone intra-articular left wrist injection. He was then evaluated by a hand surgeon within four days after onset and was thought to have gout or pseudo-gout. One week later he returned with worsening pain, swelling, and erythema around the left wrist.

Physical exam

A physical examination revealed left-hand edema, erythema, and diffuse tenderness to palpation of the wrist with surrounding warmth. No other abnormal findings were found.

Diagnostics/imaging

**Figure 2:**
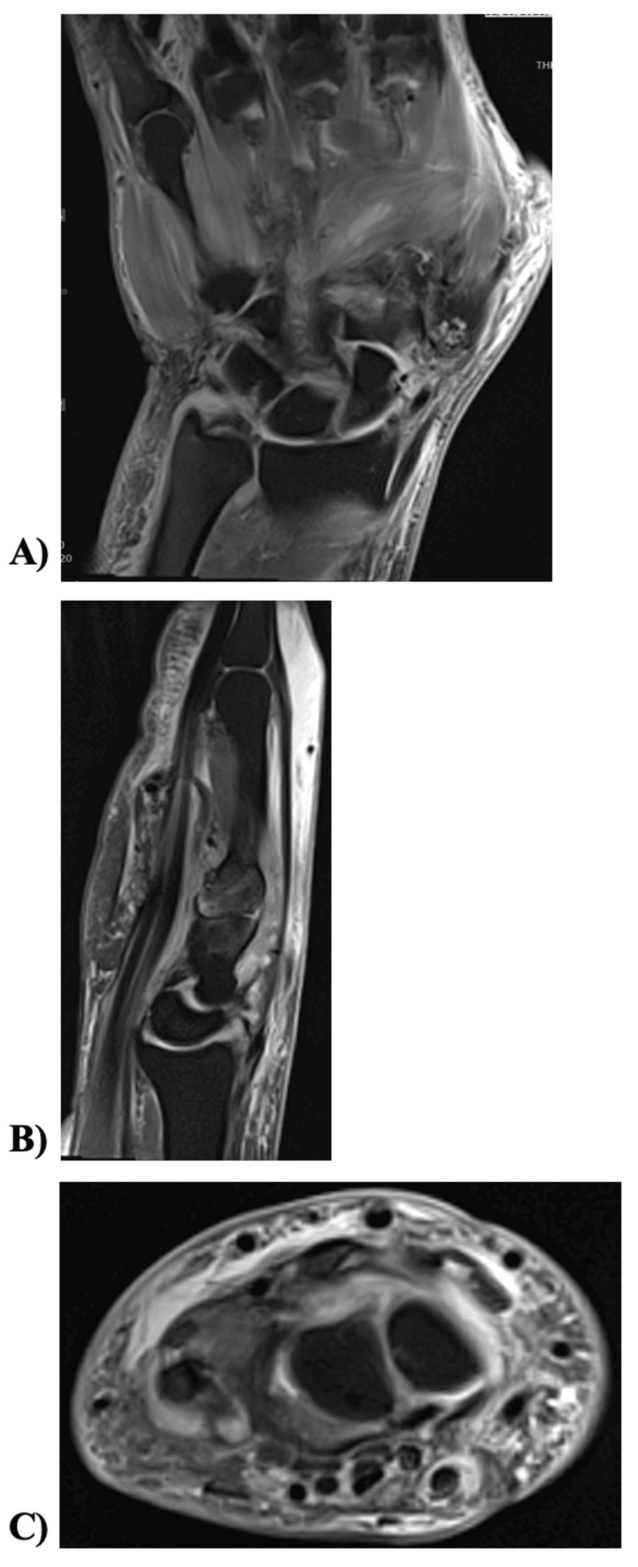
Initial left wrist MRI. (A) Coronal view showing diffuse muscle edema but no abnormal widening of the scapholunate interval or osteomyelitis. (B) Sagittal view view of the left wrist showing subcutaneous soft tissue swelling and edema throughout the hand and wrist with diffuse muscle edema. (C) Axial view showing small 14x7 mm and 7x5 mm intramuscular fluid collections in the opponens digiti minimi.

Initial imaging included radiographs with no suspicious findings. His laboratory results demonstrated leukocytosis of 12.7, ESR elevated at 30 mm/hr, and CRP elevated at 57.4. An MRI obtained one week after the initial onset showed patient underwent left wrist aspiration for presumed septic arthritis after the MRI confirmed wrist fluid (Figure 2). The aspirated fluid was not purulent. The left wrist aspirate grew *Staphylococcus aureus* susceptible to nafcillin, amoxicillin, clavulanic acid, and cephalosporins. Blood cultures obtained on admission included *Staphylococcus aureus* susceptible to nafcillin, amoxicillin, clavulanic acid, and cephalosporins. Clindamycin was confirmed by a D-test. 

Treatment

Due to positive aspiration cultures, the patient underwent a left wrist arthrotomy with synovectomy and drainage 24 hours later. Tenosynovectomy of the extensor digitorum communis tendon to the left index, middle, and ring fingers, as well as the extensor indices proprius, extensor pollicis longus, extensor carpi radialis longus, and extensor carpi radialis brevis, incision and drainage of the left palm measuring 6 sq cm, and manipulation under anesthesia of all joints in hand, fingers, and wrist were performed. Tenosynovium was collected from extensor tendons and sent for pathologic analysis, crystal analysis, and cultures. The scapholunate ligament was found to be torn, consistent with the MRI. Initial bone biopsy resulted negative. There was significant inflammatory synovitis in the wrist joint including the radiocarpal and midcarpal joints, but no obvious purulence was seen.

Infectious disease was consulted, and the patient was subsequently placed on six weeks of intravenous cefazolin and aggressive certified hand therapy (CHT).

**Figure 3:**
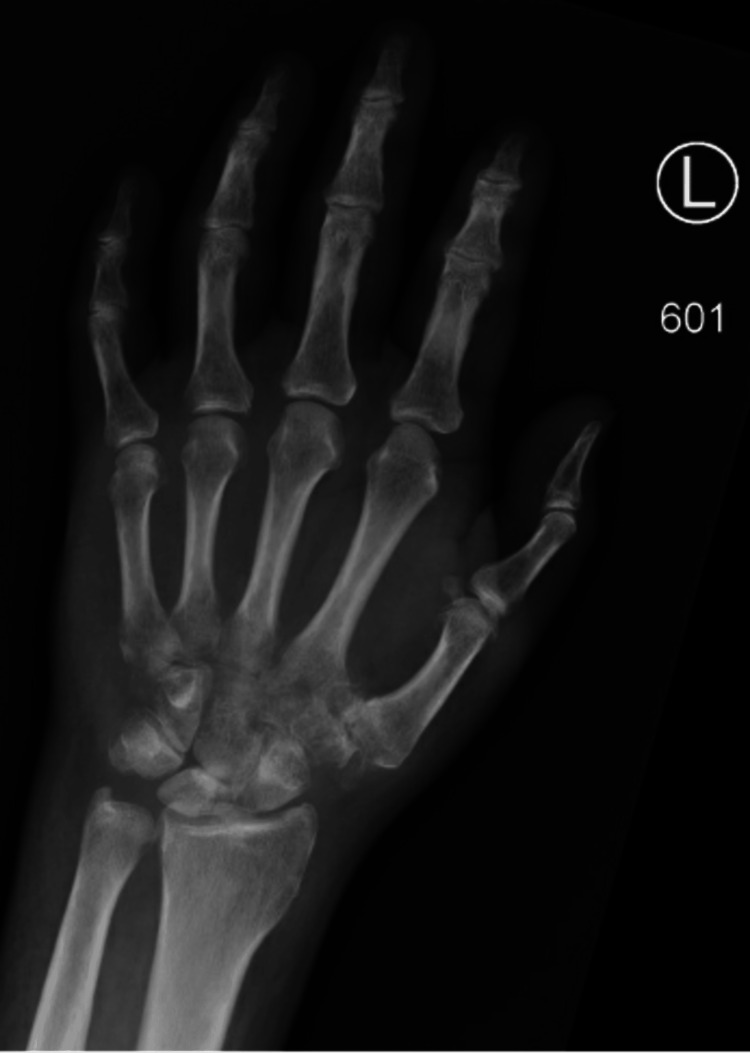
Follow-up left wrist radiograph. Radiograph, about six weeks after irrigation and debridement, showing extreme demineralization of most of the carpal bones and bases of the metacarpals, suggestive of osteomyelitis.

**Figure 4:**
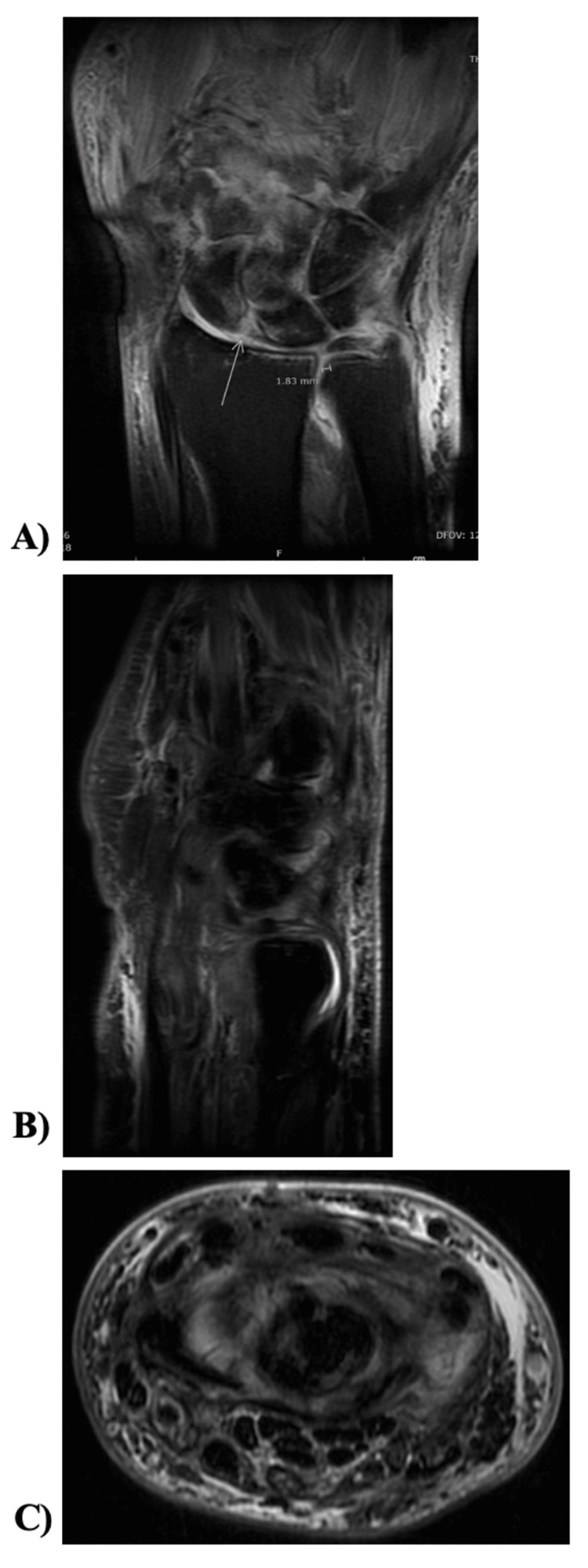
Repeat left wrist MRI after six weeks of symptoms. MRI of left wrist, six weeks after irrigation and debridement for septic arthritis. (A) Coronal, (B) sagittal, and (C) axial views showing moderate enhancing synovitis and capsulitis of the midcarpal, radiocarpal (green arrow), and distal radioulnar joints and worsening bone marrow signals of the proximal second and third metacarpals, captitate, and trapezoid with appearance of osteomyelitis.

Six weeks after surgery, the patient had mild improvement in pain and swelling; however, routine radiographs (Figure 3) demonstrated marked osteolysis of trapezoid, capitate, hamate, and proximal metacarpals two through five. A prompt repeat follow-up MRI (Figure 4) showed likely osteomyelitis including the trapezoid, capitate, hamate, and proximal metacarpals two through five.

**Figure 5:**
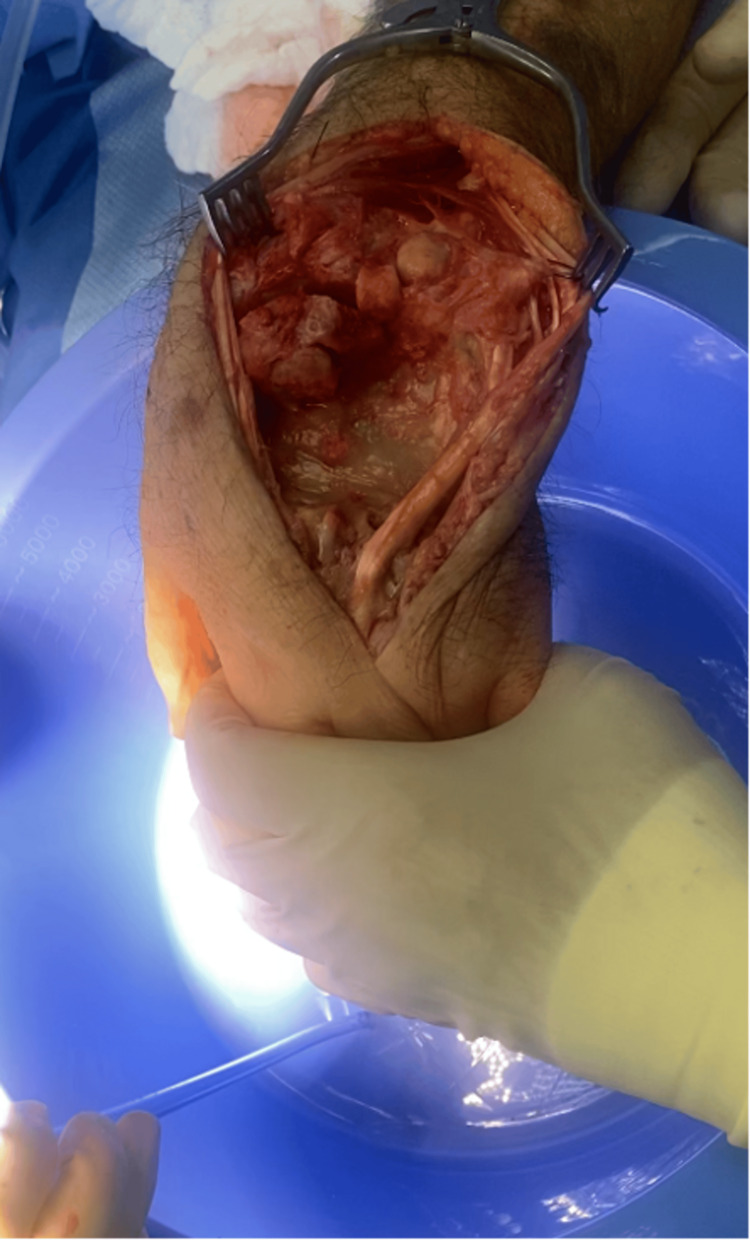
Intraoperative image of left wrist status post irrigation and debridement.

Two months after the initial presentation and six weeks from the initial washout, patient care was transferred to Dr. Hustedt with the approval of the initial hand surgeon. The patient underwent left wrist irrigation and debridement with removal of the bone. Intraoperatively, significant osteomyelitis/osteonecrosis of the proximal portions of the second, third, fourth, and fifth metacarpals, triquetrum, hamate, distal portion of the capitate, trapezoid, and trapezium was found. All infected/osteonecrotic bone was removed, including the affected metacarpals, triquetrum, hamate, capitate, trapezoid, and trapezium, followed by irrigation (Figure 5). Soft tissue and bone specimens were sent for culture. A tobramycin and vancomycin-impregnated polymethylmethacrylate (PMMA) spacer was placed under the distraction of fingers to allow maximum space occupying the cement spacer for further reconstruction. The patient was placed on intravenous cefazolin until specimen cultures resulted. Intraoperative specimen cultures grew methicillin-resistant *Staphylococcus aureus* (MRSA), and the patient was sent home on intravenous daptomycin. Oral and maxillofacial surgery was involved and ruled out a dental etiology. An echocardiogram ruled out a cardiac etiology.

The patient had an adverse reaction to daptomycin at four weeks postoperatively and was transitioned to vancomycin. He completed a six-week course of intravenous vancomycin/daptomycin and a four-week course of oral linezolid with resulting negative inflammatory markers. 

**Figure 6:**
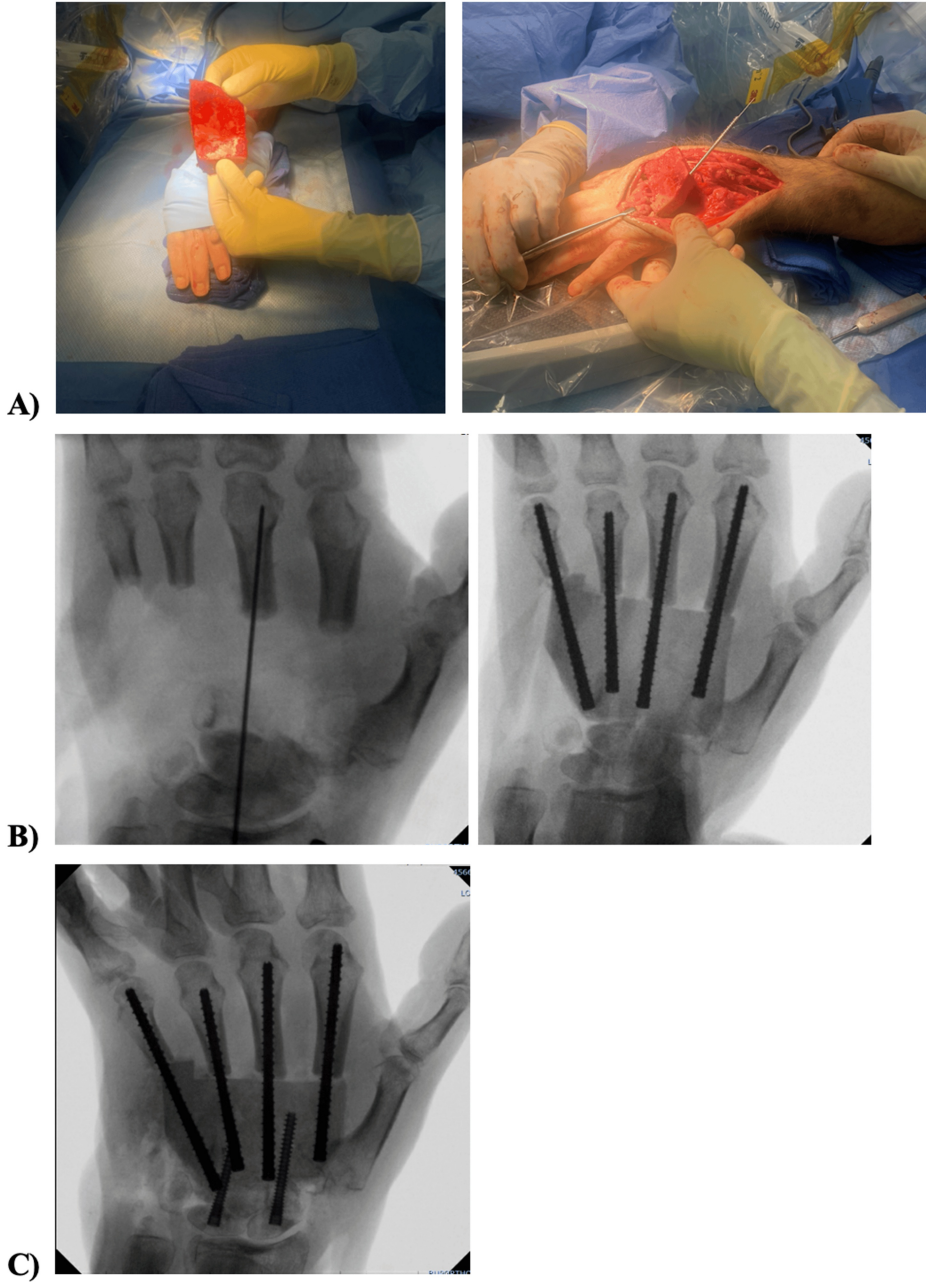
(A) Intraoperative clinical photo of iliac bone graft fixed in place with intramedullary metacarpal screws. (B) Radiographs of iliac bone graft fixed in place with intramedullary metacarpal screws. (C) Intraoperative radiograph of metacarpal intramedullary screws in second through fifth digits and screws from proximal carpal row into the iliac bone graft.

Five months after the initial presentation, he underwent reconstruction with two surgical teams. The first orthopedic team harvested the iliac crest graft as well as the right femoral canal autograft harvest from the reamer-irrigator-aspirator (RIA) for the planned Masquelet. The second orthopedic team was led by our hand surgeon, Dr. Hustedt, who reconstructed the left wrist and hand with the harvested grafts with open reduction internal fixation of left second, third, fourth, and fifth metacarpals with carpal fusion with iliac bone graft using four ExsoMed screws (Exsomed, Aliso Viejo, USA) and two Acumed Acutrak screws (Acumed Inc., Beaverton, USA) (Figure 6).

Follow-up

Postoperatively, the patient was unable to walk without assistance. He was transferred to a rehab facility for 10 days and subsequently participated in outpatient CHT. Follow-up visits showed a well-healed wound and radiographs showed appropriate healing without recurrence of infection.

Five months after reconstruction, the patient underwent percutaneous release of the second and third metacarpal joints with a passive range of motion under anesthesia. After release, the patient continued to undergo CHT without functional improvement. Seven months postoperatively since reconstruction, a one-time triamcinolone steroid injection was administered into the second and third left metacarpophalangeal joint (MCP) and proximal interphalangeal joint (PIP) on the patient's request, followed by further CHT. As a result, the patient noticed significant cutaneous fragility and atrophy three months after injection. Therapy has been halted since that time to allow the skin to recover.

**Figure 7:**
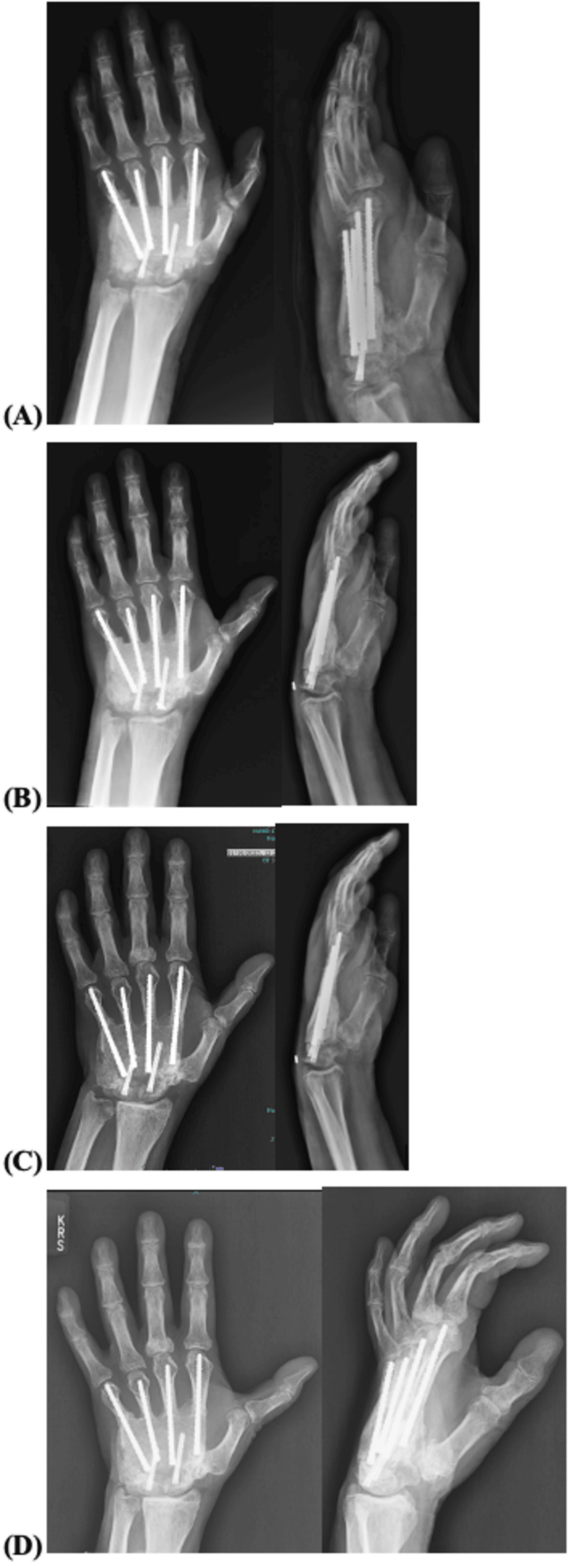
Anterior-posterior and lateral views of left-hand radiographs. Radiographs at (A) one month, (B) three months, (C) six months, and (D) one year after reconstructive surgery showing stable fusion of the graft.

The patient is now two years out from the initial presentation and one year and six months out from reconstruction. Radiographically, the reconstruction is stable (Figure 7). He continues to have left second, third, fourth, and fifth-digit MCP and PIP stiffness. He has achieved 45 degrees of flexion at the MCP. He is unable to oppose left thumb to any finger.

## Discussion

Comorbidities such as chronic kidney disease, diabetes, alcoholism, active intravenous drug use, and any condition or medication resulting in an immunosuppressed state should increase suspicion of septic arthritis [8,10]. While our patient was not immunocompromised, he did have nummular eczema, a chronic inflammatory disease, that most often presents idiopathically in males between the ages of 50-65 years [11,12]. Nummular eczema is known to cause staphylococcal colonization around the skin that is affected. The extra presence of staphylococcal species on the skin can result in a higher susceptibility rate of contracting a staphylococcal infection. To reduce the inflammatory symptoms that arise from nummular eczema, topical cortisone ointments are often prescribed to patients as prescribed for this patient [10].

This case emphasizes how easily septic arthritis can be overlooked and misdiagnosed. Studies have shown a good prognosis if surgical drainage can occur in the affected joint within 10 hours of diagnosis and negative sequelae when surgical intervention is delayed beyond 16 hours [2]. Delayed presentation has an increased risk of leading to mortality. Yap and Tay showed a 15% mortality rate six months after presentation, with a mean time of 6.8 days from presentation to surgery [8].

In addition, we would like to highlight the use of a Masquelet technique for surgical treatment. The Masquelet technique is typically performed as a two-staged approach. It was first described in treatment of lower extremity long bone defects [13]. As was exhibited in this case, the first stage involves debridement followed by a cement stabilizer. The second stage typically occurs within four to six weeks from the first stage, which allows for the pseudo-membrane to form, at which time the spacer is replaced with the cancellous bone graft [14]. The success of Masquelet technique is credited to the vascularized pseudo-membrane that produces various growth factors including bone morphogenic protein 2 (BMP2), transforming growth factor beta (TFG-β), and vascularized endothelial growth factor (VEGF) [15,16]. Historically, Masquelet has been taught to be the method of treatment for bone defects in the upper extremity that are smaller than 6 cm, while vascularized bone graft was the preferred method for bone defects greater than 6 cm. However, systematic reviews have evaluated the “6 cm rule” and determined that there is no strong evidence to support that vascularized bone graft is more effective than Masquelet for defects greater than 6 cm or even as large as 10 cm [17-19].

## Conclusions

In summary, this case is an example of successful management of osteomyelitis involving the left trapezoid, capitate, hamate, and proximal two through five metacarpals in a 68-year-old, right-hand dominant hand surgeon. The overall technique included a wide excision with concurrent long-term antibiotics, followed by a reconstruction using the right iliac cortical autograft, combined with Masquelet over a six-month course.

Our case highlights several important teaching points. First, the early diagnosis of septic arthritis requires clinical suspicion based on the salient features that can be recognized from a combination of clinical history, aspiration results, and laboratory results. Recognition allows hand surgeons to properly address brewing infection and possibly prevent detrimental morbidity through progressing disease. Second, clinicians should be suspicious of septic arthritis in the setting of an immunocompetent adult who has increased risk through skin conditions, such as nummular eczema that requires steroid treatment. Third, in patients like our case who have progressed wrist osteomyelitis with large defects, the Masquelet technique as a two-staged procedure can be effective in treatment of large defects, above 6 cm, of the upper extremity.
